# Complications caused by adenosine during catheter ablation of atrial fibrillation

**DOI:** 10.1002/ccr3.906

**Published:** 2017-04-04

**Authors:** Jackson J. Liang, Ramanan Kumareswaran, Matthew C. Hyman, Roger A. Moore, David Lin, David S. Frankel

**Affiliations:** ^1^ Cardiovascular DivisionElectrophysiology SectionHospital of the University of PennsylvaniaPhiladelphiaPennsylvania; ^2^ Department of AnesthesiologyHospital of the University of PennsylvaniaPhiladelphiaPennsylvania

**Keywords:** Adenosine, anaphylaxis, atrial fibrillation, bronchospasm, catheter ablation

## Abstract

Adenosine is increasingly used to assess for dormant conduction following pulmonary vein isolation during atrial fibrillation ablation. While the half‐life of adenosine is typically short and side effects transient, operators should be aware of more serious, lasting adverse reactions including anaphylaxis and bronchospasm.

## Introduction

Adenosine is frequently given during atrial fibrillation (AF) ablation to assess for dormant conduction following pulmonary vein isolation (PVI). Acute effects of adenosine include hypotension and bronchospasm, both of which typically resolve within seconds due to adenosine's short half‐life. We describe one case of prolonged hypotension due to anaphylaxis and another of prolonged bronchospasm‐induced hypoxia. With increasing use of adenosine during catheter ablations, operators should be aware of these uncommon but important adverse reactions.

## Case 1

A 57‐year‐old man with hypertension, treated with lisinopril and metoprolol succinate, and symptomatic, paroxysmal AF underwent catheter ablation. A radial arterial line was inserted for hemodynamic monitoring. General anesthesia was induced with 50 μg fentanyl, 100 mg succinylcholine, and 200 mg propofol prior to intubation, then maintained with propofol 75 μg/kg/min and remifentanil 0.15 μg/kg/min. Blood pressure was supported with phenylephrine 75 μg/min. High‐frequency, low volume (JET) ventilation was utilized to enhance catheter stability. The pulmonary veins were isolated uneventfully.

**Figure 1:**
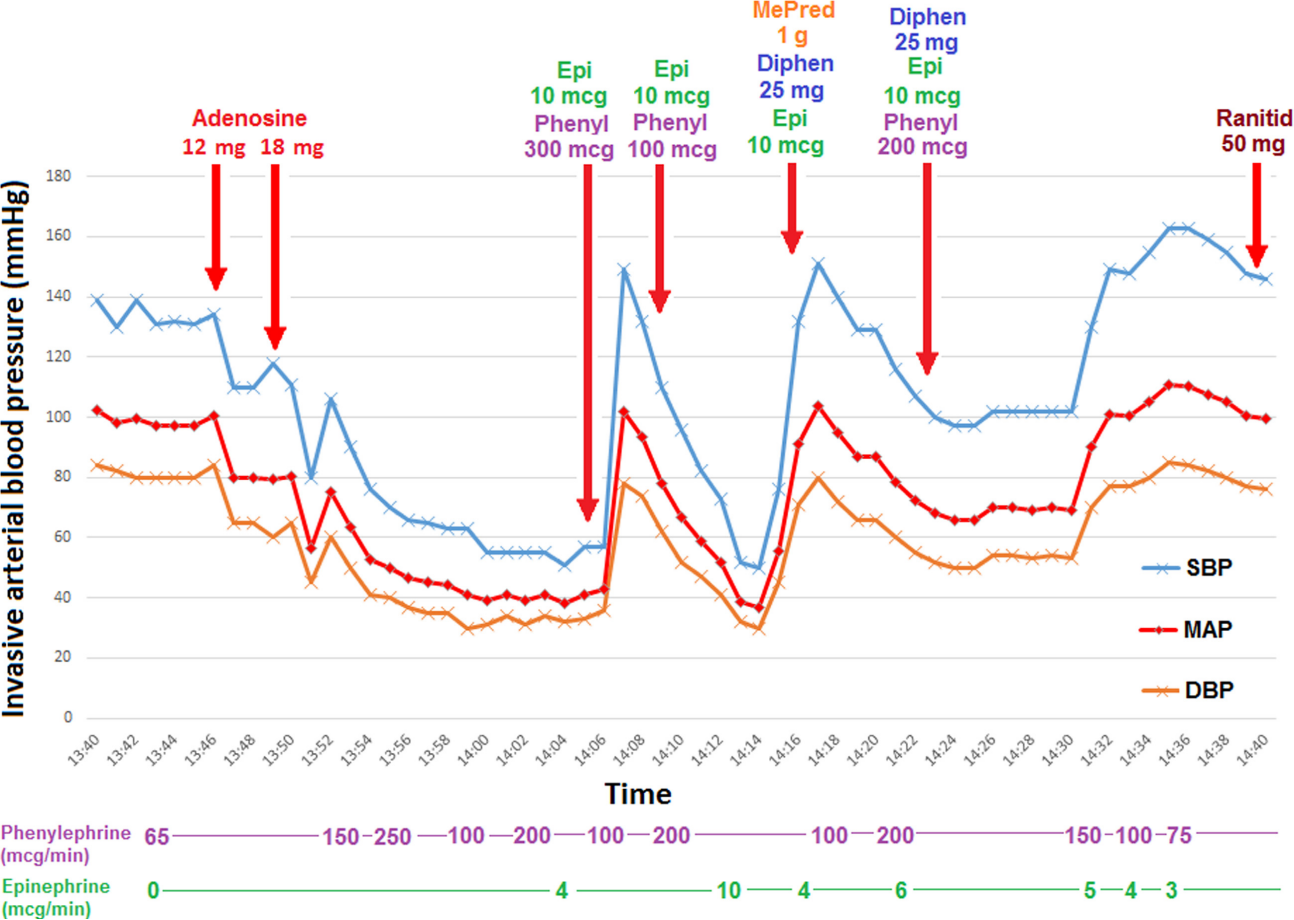
Invasive arterial blood pressure trend following administration of adenosine. Blood pressure trend demonstrating acute onset of sustained hypotension immediately following administration of intravenous adenosine (12 mg then 18 mg). Blood pressure recovered after 45 min of fluid resuscitation, as well as administration of epinephrine, phenylephrine, methylprednisolone, diphenhydramine, and ranitidine. Bolus doses are displayed above the graph and continuous infusion rates below. Epi, epinephrine; Phenyl, phenylephrine; MePred, methylprednisolone; Diphen, diphenhydramine; Ranitid, ranitidine; SBP, systolic blood pressure; DBP, diastolic blood pressure; MAP, mean arterial pressure.

Following PVI, the patient's blood pressure was 135/85 mmHg. Adenosine 12 mg was administered as a rapid bolus via a central venous line with no prolongation of the PR interval. Repeat blood pressure after 2 min was 110/60 mmHg. Adenosine 18 mg was administered next, prolonging the PR interval from 155 to 230 msec, following which the blood pressure precipitously decreased to 55/30 mmHg (Fig. 1) and facial flushing developed. Remifentanil was discontinued, yet he remained profoundly hypotensive requiring epinephrine (40 μg via 4 boluses, followed by infusion at 10 μg/min), phenylephrine (600 μg via 3 boluses, followed by infusion at 200 μg/min), and multiple boluses of normal saline to maintain a mean arterial pressure >50 mmHg. There was no pericardial effusion by intracardiac echocardiogram (ICE), and point‐of‐care hemoglobin was unchanged. Due to concern for anaphylactic reaction, intravenous methylprednisolone (1 g), diphenhydramine (50 mg), and ranitidine (50 mg) were administered, in addition to the epinephrine. After 45 min of hypotension, the flushing resolved and his blood pressure recovered to his previous requirement of phenylephrine 75 μg/min. He was awakened from general anesthesia with a normal neurological examination. Abdominal CT scan demonstrated no retroperitoneal bleed. The patient was monitored overnight and discharged the following day with his usual blood pressure.

## Case 2

A 52‐year‐old woman with obesity and symptomatic, persistent AF refractory to propafenone and flecainide underwent catheter ablation. She was a nonsmoker without history of pulmonary disease. She was placed under general anesthesia and mechanically ventilated using JET. After isolating the pulmonary veins, 18 mg of adenosine was administered. Atrioventricular block and acute reconnection of the left superior PV were observed. Immediately following adenosine administration, the peak inspiratory pressure increased and oxygen saturation decreased from 99% to 78%. Auscultation demonstrated bilateral expiratory wheeze. Albuterol was administered with subsequent improvement in ventilation and recovery of oxygenation. Additional ablation lesions were delivered at the site of acute reconnection, following which 18 mg of adenosine was administered a second time. Again, she immediately developed bronchospasm, with increased peak inspiratory pressures and decreased oxygen saturation, which resolved within minutes following albuterol administration. The procedure was successfully concluded without further administration of adenosine.

## Discussion

Adenosine has a short half‐life and is typically given in low doses (6 or 12 mg). Well‐known side effects of adenosine include chest pressure, facial flushing, and headache. Despite mixed results of published studies, adenosine is increasingly administered to assess for dormant conduction following PVI 1, 2, 3, 4. Further, in the context of AF ablation, higher doses of adenosine (18 or 24 mg) are often required to affect atrioventricular conduction, particularly in obese patients 5. Given increasing utilization of high doses of adenosine during AF ablation procedures, electrophysiologists should be aware of more serious adverse reactions including anaphylaxis and severe bronchospasm. We describe two cases illustrating these infrequent, but serious reactions.

### Hypotension

The differential diagnosis for acute hypotension during AF ablation includes cardiac tamponade, vascular bleeding, pulmonary embolism, and medication induced. In our patient (Case 1), ICE rapidly excluded cardiac tamponade, the groins were soft, and hemoglobin was unchanged. CT scan of the abdomen and pelvis later ruled out bleeding entirely.

Administration of protamine sulfate is well known to cause hypotension 6. We present a prolonged episode of hypotension in response to adenosine, lasting long after AV conduction recovered. The half‐life of adenosine is short (sec) as it is rapidly taken up by erythrocytes and vascular endothelial cells and inactivated intracellularly, either via phosphorylation by adenosine kinase to adenosine monophosphate (AMP) or by deamination to inosine via adenosine deaminase. Therefore, patient 1's prolonged hypotension was highly unusual and presumed to be secondary to anaphylaxis, given the persistence of hypotension and associated flushing. Hypotension resolved following administration of epinephrine, diphenhydramine, and ranitidine. Anaphylactic reaction to adenosine has been previously reported 7.

### Hypoxia

The differential diagnosis of acute hypoxia during AF ablation includes endotracheal tube dislodgement, mucus plug, pulmonary edema, and pulmonary embolism. When occurring immediately after adenosine administration, one should suspect adenosine‐induced bronchospasm, a well‐described adverse effect 8, 9, 10. Treatment includes inhaled bronchodilators such as albuterol. Additionally, inhaled anesthetic agents, such as sevoflurane, isoflurane, and halothane, may also mitigate bronchospasm.

## Conclusion

With adenosine being increasingly used to assess for dormant conduction following pulmonary vein isolation, operators should be aware of infrequent yet serious adverse reactions, including prolonged hypotension from anaphylaxis, and severe bronchospasm. Prompt recognition will allow for timely treatment and avoidance of unnecessary diagnostic testing.

## Authorship

JJL, RK, MCH, RAM, DL, and DSF: all contributed to the preparation, review, and submission of the manuscript.

## Conflict of Interest

The authors report no relevant conflict of interests.
